# Control and mitigation of dengue and Zika virus transmission in a hospital in Recife, Brazil: a successful experience with an integrated control program against *Aedes aegypti*

**DOI:** 10.1186/s13071-025-07241-9

**Published:** 2026-03-13

**Authors:** Helena Emanuela Candida-Silva, Henrique Rafael Pontes Ferreira, Jaziela de Arruda Mendonça, Rafael Alves da Silva, Larissa Krokovsky, Letícia de Oliveira Martins, Kathyanne Ellen da Silva Barbosa, Frederico Jorge Ribeiro, Cláudia Maria Fontes de Oliveira, Constância Flávia Junqueira Ayres, Marcelo Henrique Santos Paiva, Maria Alice Varjal de Melo-Santos

**Affiliations:** 1 Departamento de Entomologia, Fundação Oswaldo Cruz (FIOCRUZ), Instituto Aggeu Magalhães, Campus da Universidade Federal de Pernambuco, Cidade Universitária, Av. Professor Moraes Rego S/N, Recife, Pernambuco CEP: 50670420 Brasil; 2 Hospital das Clínicas, UFPE/EBSERH, Recife, Pernambuco Brasil; 3 Núcleo de Ciências da Vida, Universidade Federal de Pernambuco, Caruaru, Pernambuco Brasil

**Keywords:** Arbovirus, Nosocomial infections, Vector surveillance, Mosquito control

## Abstract

**Background:**

The presence of *Aedes aegypti* in healthcare facilities represents a significant epidemiological threat, as it is the primary vector of dengue (DENV), Zika (ZIKV), and chikungunya (CHIKV) viruses. In the absence of effective alternatives to chemical insecticides for eliminating adult mosquitoes, healthcare facilities may become focal points for arbovirus transmission, compromising the safety of patients and healthcare employees. Vector control remains the main approach for mitigating arbovirus transmission. This study investigated the impact of an effective integrated control program (ICP) implemented in a hospital in Recife, Pernambuco, Brazil, where arbovirus circulation in mosquitoes was detected.

**Methods:**

Entomological monitoring was conducted through mechanical aspiration inside the Hospital das Clínicas (HC). Specimens were counted, identified, grouped by species, classified according to blood-feeding status, and recorded by capture station. Viral infection was assessed using nonstructural protein 1 (NS1) by enzyme-linked immunosorbent assay (ELISA) and a triplex reverse transcription quantitative polymerase chain reaction (RT-qPCR) for the detection of DENV, ZIKV, and CHIKV. Infection rates were calculated using the minimum infection rate (MIR). ICP actions included environmental management, larvicide application at breeding sites, ovitraps to collect and destroy eggs, toxic sugar baits, and intensified mechanical aspiration to eliminate adult mosquitoes. Three phases were defined: pre-ICP (initial survey), ICP (all actions), and post-ICP (larvicidal treatment).

**Results:**

During the ICP, a sustained reduction in female mosquito density was observed, reaching 99% for *Ae. aegypti* and 88% for *Culex quinquefasciatus*. Six months after ICP, ELISA detected infected females in 9 of 14 stations (64%). The MIR for *Ae. aegypti* decreased from 50 to 44, while *Cx. quinquefasciatus* showed a slight increase. RT-qPCR revealed 11 ZIKV-positive pools (eight *Ae. aegypti* and three *Cx. quinquefasciatus*). After 12 months of ICP, only one *Ae. aegypti* DENV-positive pool was detected by both techniques in a single station (pediatrics). Both techniques detected infection even in pools containing a single female, underscoring their high sensitivity. At 18 months of ICP, all pools tested negative for arboviruses.

**Conclusions:**

Molecular and antigen-based approaches confirmed the mitigation of arbovirus circulation in hospital-associated mosquitoes, demonstrating that the continuous elimination of *Ae. aegypti* females (> 99%) is critical in these settings to prevent nosocomial transmission.

**Graphical abstract:**

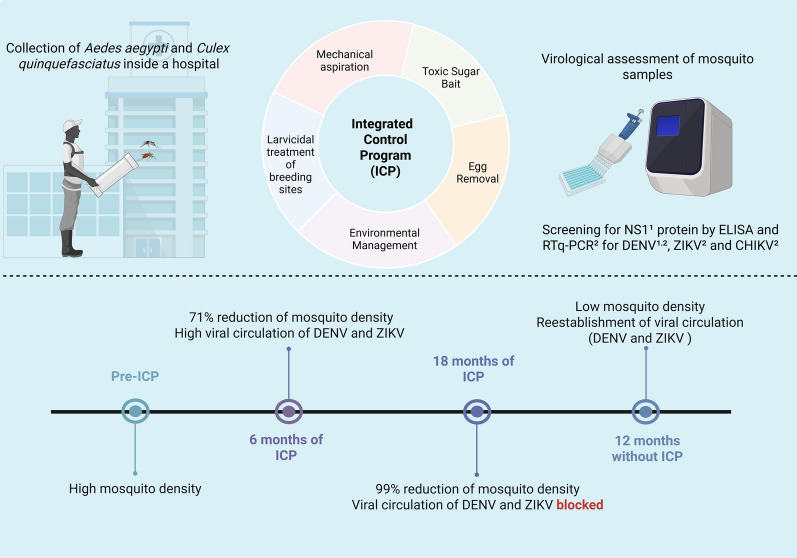

**Supplementary Information:**

The online version contains supplementary material available at 10.1186/s13071-025-07241-9.

## Background

Every year, thousands of Brazilian citizens presenting with clinical symptoms of arboviral infections seek care through the Unified Health System (*Sistema Único de Saúde*—SUS), in particular at public healthcare facilities such as Basic Healthcare Units (*Unidades Básicas de Saúde*—UBS) and Emergency Care Units (*Unidades de Pronto Atendimento*—UPA), which constitute primary healthcare services. Polyclinics and reference hospitals receive patients with more severe arbovirus-associated complications [1]. Dengue virus (DENV) has followed an endemic-epidemic pattern in Brazil since the 1980s [2]; however, in 2024, the country experienced its largest outbreak, with more than 6.6 million cases reported across several states [3]. Over the past decade, Brazil has faced a historically widespread increase in arboviral diseases, associated with the spread of Zika virus (ZIKV), chikungunya virus (CHIKV), yellow fever virus, and, more recently, Oropouche virus (OROV) [4, 5].

Sequelae associated with CHIKV, ZIKV, and OROV infections have been linked to immunological alterations that may trigger autoimmune diseases, such as Guillain–Barré syndrome, as well as neurological complications, including congenital Zika syndrome (CZS), microcephaly, and other conditions [6], which remain important public health concerns. In Brazil, the epicenter of CZS- and ZIKV-associated microcephaly cases was in the state of Pernambuco, particularly in the city of Recife, where the highest number of affected children in the country was recorded [6, 7].

Beyond the spread of arboviruses in urban areas, certain environments, such as hospital settings, are more sensitive to the transmission of pathogens, including multidrug-resistant bacteria and fungi [8–10]. The presence of *Aedes aegypti* mosquitoes in these environments may represent a significant epidemiological risk for nosocomial arbovirus transmission [10–13]. Previous study conducted at hospitals and local health units from the Recife Metropolitan Region (RMR) [13] reported the circulation of ZIKV and DENV in both *Ae. aegypti* and *Culex quinquefasciatus*, providing baseline evidence of viral circulation in these facilities. This scenario is especially concerning because these locations also provide elective care for immunocompromised patients, pregnant women, and an aging population with comorbidities or in vulnerable situations [8–11, 13].

Strategies to mitigate arboviral transmission remain closely associated with the effectiveness of vector control [14–16], given the limited availability of vaccines within SUS [17]. Surveillance and control of *Ae. aegypti* continues to represent major challenges for health services worldwide. In this context, the integrated vector management (IVM) framework established by the World Health Organization promotes evidence-based, intersectoral, and sustainable approaches to vector control. This strategy emphasizes the integration of entomological surveillance, environmental management, community engagement, and policy support to reduce disease transmission [18].

The National Dengue Control Program (*Programa Nacional de Controle da Dengue*—PNCD), launched in Brazil in 2002, has implemented actions aimed at eliminating larval breeding sites through mechanical removal or environmental management or through treatment with chemical and biological larvicides at bimonthly intervals. In addition, biweekly spatial applications (ultralow-volume) of chemical insecticides are recommended for strategic places such as cemeteries, junkyards, tire shops, recycling sites, and others, to eliminate adult mosquitoes, or in areas of active viral transmission [19].

This challenge is particularly evident in tropical cities such as Recife, the capital of Pernambuco in northeastern Brazil, where environmental and infrastructural conditions favor viral co-circulation and high mosquito densities. These conditions include large urban agglomerations and areas with deficiencies in water supply and sanitation systems, consistently high temperatures (25–30 °C), and elevated precipitation levels [20], especially during the rainy season from March to August [8, 9, 11–13]. Such conditions sustain local *Ae. aegypti* infestation alongside other mosquito species, including *Cx. quinquefasciatus*, which in urban areas of the RMR has been observed to occur in even greater numbers than *Ae. aegypti* in intradomiciliary environments [21].

Among vector monitoring strategies, RT-qPCR is considered the gold standard for arbovirus detection in mosquitoes due to its high sensitivity and specificity [22, 23]. This technique has the advantage of detecting viral RNA particles. However, it also presents limitations that restrict the routine inclusion of entomo-virological surveillance in the PNCD. These limitations are mainly associated with the need for a continuous cold chain, including storage in ultralow-temperature freezers (−80 °C), for the preservation and transportation of mosquito samples collected in the field [24, 25].

In extreme cases, when mosquito samples are poorly preserved or RNA integrity is compromised, antigen detection methods such as enzyme-linked immunosorbent assay (ELISA), targeting the nonstructural protein 1 (NS1) antigen, have long been used to detect arboviruses such as DENV in humans and have proven to be an option for virological surveillance in mosquitoes [26–29]. NS1 is a highly conserved protein secreted by infected cells during the early phase of viral replication and can be detected in both human serum and mosquito homogenates [26, 27]. Commercial ELISA kits have been successfully employed to identify DENV infections in *Ae. aegypti* [26–29].

In this context, our study demonstrates the mitigation of nosocomial transmission risk for DENV, ZIKV, and CHIKV through the implementation and successful execution of an integrated control program (ICP) targeting *Ae. aegypti* in a public hospital in Recife, Pernambuco, Brazil. The reduction of vector-borne transmission was confirmed using two independent diagnostic approaches: NS1 antigen detection and RT-qPCR.

## Methods

### Study area

The Hospital das Clínicas (HC) is affiliated with the Universidade Federal de Pernambuco (UFPE) and the Empresa Brasileira de Serviços Hospitalares, located in Recife, Brazil, and is a high-complexity healthcare facility integrated into the SUS. It stands out as a reference hospital in 42 medical specialties, including oncology, nephrology, high-risk pregnancy, and neurology. According to the 2023 Management Report of the HC Professor Romero Marques, UFPE (2018/2019), HC handles a substantial patient volume, averaging approximately 10,000 consultations, 15,000 diagnostic examinations, more than 500 surgical procedures, and 500 hospitalizations per month. The hospital’s population includes approximately 3,000 people involved in its activities, in addition to the thousands of patients and their companions who visit the institution each month. This intense human activity, combined with the large number of patients, highlights the hospital’s crucial role as a healthcare and educational hub, while also representing an environment with high risk of infections. 

The HC comprises a built area of approximately 62,000 m^2^, with horizontal and vertical buildings (11 floors), including waiting areas, offices, surgical blocks, beds, a restaurant, kitchen, dining room, laundry, mechanical/electrical workshops, and a morgue. HC has a basement area with 12 drainage tanks and eight elevator shafts, as well as changing rooms and bathrooms for employees.

This facility was selected for the present study based on previous findings indicating a critical situation of *Ae. aegypti* infestation and arbovirus detection in field-collected samples from 2014/2015 [12, 13].

### Experimental design

**Fig. 1:**
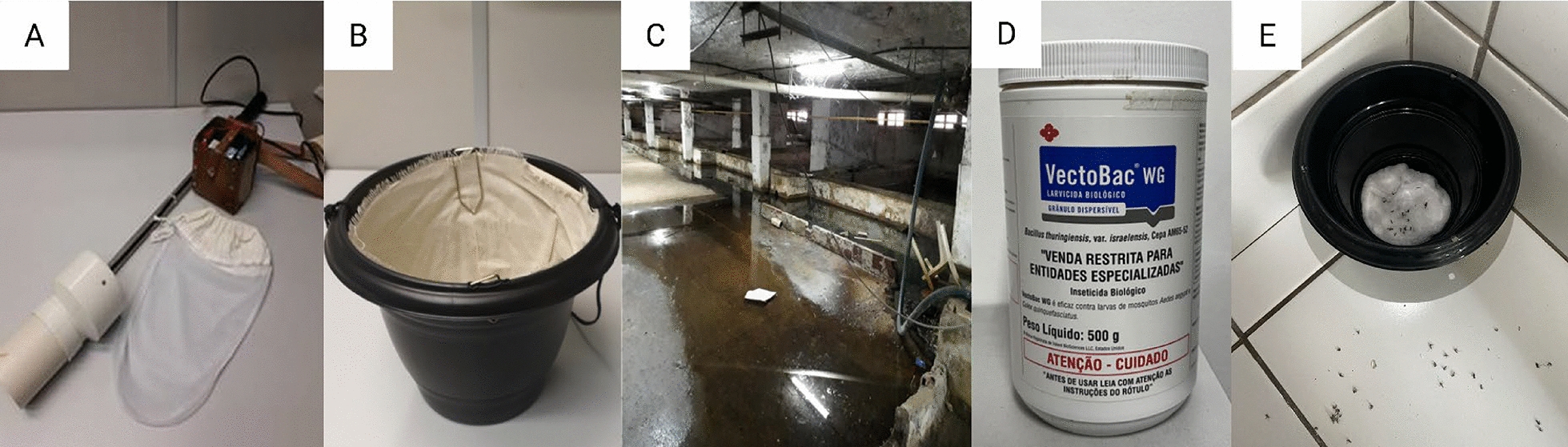
Mosquito monitoring and control tools used at the Hospital das Clínicas: **A** battery-powered entomological aspirator with collection bag; **B** control ovitrap; **C and D** drainage tanks treated with the larvicide *Bacillus thuringiensis israelensis* (Bti); **E** prototype of a toxic sugar bait treated with ivermectin (TSB_Ivermec_) showing dead mosquitoes inside and around the device

**Fig. 2:**
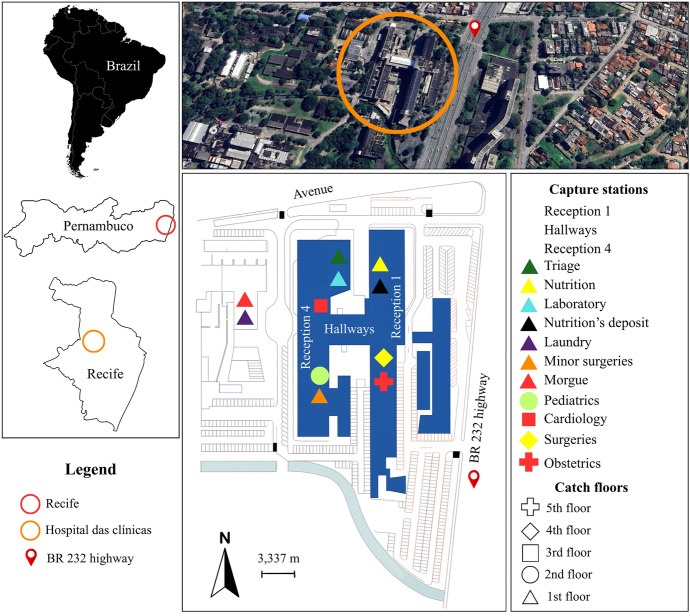
Capture stations for mechanical aspiration of adult mosquitoes at the Hospital das Clínicas, Federal University of Pernambuco (UFPE), during different phases of the integrated mosquito control program (August 2018 to March 2021)

This is a quasi-experimental before-and-after study, without randomization, that describes the specific impact of an ICP against *Ae. aegypti* in reducing the vectorial risk of arbovirus transmission. Mosquito monitoring and control tools used at the HC are shown on Fig. 1. The investigation was carried out at 14 fixed points within the HC, referred to as capture stations (Fig. 2), from August 2018 to March 2021 to quantify infected females across three different periods, as described below.

- Pre-ICP—in August 2018, a pre-survey of mosquitoes presence inside the HC was conducted, using mechanical aspiration of the specimens in resting places.
- ICP—September 2018 to February 2020, when multiple control strategies were implemented to reduce mosquito infestation, including environmental management, capture and mechanical removal of eggs using 50 control-ovitraps (C-OVT), biological larvicidal treatment of permanent breeding sites located in the basement and outdoor areas, and finally the mechanical removal of adults by aspiration and their elimination through the use of 150 ivermectin toxic sugar bait (TSB_Ivermec_) prototypes.
- Post-ICP—March to September 2020, when all control strategies were interrupted due to the COVID-19 pandemic. After that, from October 2020 to February 2021, the control actions were reduced to exclusively larvicidal treatment of breeding sites.

The impact of the ICP was analyzed at six time points: pre-ICP; 6, 12, and 18 months during ICP; and 7 and 12 months post-ICP. Details of the control actions, including the monitoring tool (aspiration) for *Ae. aegypti*, are provided in Table 1.

**Table 1:** Monitoring and control actions for *Aedes aegypti* at Hospital das Clínicas (HC), Recife, Pernambuco, Brazil, during different periods of the integrated control program (ICP) (August 2018 to March 2021)

Period	Type of action	Tool	Location	Maintenance frequency	Number
All	Adult monitoring	Mechanical aspiration	Indoor area	Monthly	14 stations
ICP	Mechanical-control	Environmental management^b^	Basement and outdoor area	2/year	NA
ICP	Egg control	C-OVT^c^	Basement and outdoor area	Biweekly	50
ICP	Larvae control	Bti biolarvicide^d^	Basement and outdoor area	Biweekly/monthly	12/12
ICP	Adult control	TSB_Ivermec_^e^	Indoor area	Biweekly	150
ICP	Adult control	Mechanical aspiration	Indoor area	3 times/week	14 stations
Post-ICP^a^	Larvae control	Bti biolarvicide^d^	Basement and outdoor area	Monthly	12

^a^Post-intervention control program; NA: Not Analyzed

^b^Environmental management: solid waste collection and water drainage

^c^Control-ovitrap: egg capture

^d^Bti biolarvicide: breeding site treatments with *Bacillus thuringiensis israelensis*

^e^TSB_Ivermec_ = toxic sugar bait with ivermectin to kill adults mosquitoes

### Female mosquito sampling

Adult mosquitoes were captured indoors at resting places in the 14 established stations within the HC between August 2018 and March 2021. Entomological aspirators (Horst Armadilhas^®^), consisting of a polyvinyl chloride (PVC) tube connected to a 12-V battery and an inverted fan, were used to aspirate mosquitoes into an entomological bag (Fig. 1A).

Collections were conducted in the afternoon (3:00–5:00 pm) for approximately 15 min per station. Aspiration was performed on surfaces such as ceilings and floors and behind hospital furniture. The entomological bags were taken to the Entomology Department Laboratory at Fundação Oswaldo Cruz (FIOCRUZ), Pernambuco, where mosquitoes were anesthetized by cooling, identified using a dichotomous key [30], counted, and separated by capture station and sex.

Females were classified as blood-fed (BF) or non-blood-fed (NBF) based on the presence of blood in the abdomen, according to the Sella scale [31]. In this case, NBF may correspond to females that have not yet fed on blood or those that had just digested a blood meal. The BF females were also categorized according to the estimated time required to complete blood digestion (≤ 3 days and ≥ 3 days). After classification, mosquitoes were frozen for subsequent analyses.

The females were grouped by species and by feeding status (NBF or BF, regardless of the blood digestion stage) and separated into pools of up to 10 individuals for posterior analyses. Pools were macerated in 300 µl of L-15 medium supplemented with 5% fetal bovine serum (FBS) using sterile micropestles to obtain a homogenate suitable for both immunological and molecular analyses. Aliquots of the homogenates were subsequently used for the detection of viral antigens by ELISA and RNA by multiplex RT-qPCR.

### NS1 detection using the Panbio™ Dengue Early ELISA Kit

Mosquito pools collected during the pre-ICP and post-ICP periods were retrieved from storage at −30 °C. In contrast, the pools collected at different time points during the ICP itself—6 months (February 2019), 12 months (August 2019), and 18 months (February 2020)—were stored at −80 °C for NS1 antigen and molecular analysis.

All female pools were screened for NS1 antigen using the Panbio™ Dengue Early ELISA kit (Abbott Laboratories) according to the manufacturer’s instructions, with minor adaptations for mosquito samples. From each previously prepared homogenate (300 μl in L-15 medium with 5% FBS), a 75 μl aliquot was directly used for NS1 detection. Absorbance was measured at 450 nm, with 620 nm as the reference wavelength. A calibrator, a positive control, and a negative control provided with the kit were included on each plate for quality control. Results were interpreted according to the manufacturer’s criteria.

### Detection of DENV, ZIKV, and CHIKV by multiplex RT-qPCR

RT-qPCR was conducted with the same pools processed by the NS1 rapid test. For RNA extraction, 100 μl of each homogenate was used following the TRIzol (Invitrogen) protocol. Viral detection of DENV, ZIKV, and CHIKV was performed by multiplex RT-qPCR using validated primers and probes [32–34] in a QuantStudio 5 Real-Time PCR System (Applied BioSystems, Waltham, MA, USA). Reactions were prepared with the QuantiNova Probe RT-PCR Kit (Qiagen, Hilden, Germany) in a final volume of 10 µl, containing 5.0 µl of 5× Master Mix, 0.1 µl of RT Mix, 0.05 µl of ROX (5-carboxy-X-rhodamine) reference dye, 0.08 µl of each primer (800 nM), 0.04 µl of each probe (100 nM), 3.5 µl of RNA sample, and nuclease-free water to complete the volume. Cycling conditions consisted of reverse transcription at 45 °C for 15 min, initial denaturation at 95 °C for 5 min, and 45 cycles of 95 °C for 5 s and 60 °C for 45 s. The samples were tested in duplicate and accompanied by appropriate controls, including negative controls (containing all reagents except RNA and a negative extraction control), and positive controls derived from a standard curve. The standard curve was constructed according to Kong et al. [35], and consisted of five serial dilution points, each containing a defined number of RNA molecules.

Results were analyzed using QuantStudio Design & Analysis software 1.3.1 (Thermo Fisher Scientific) with automatic baseline and threshold settings, and samples with quantification cycle (C_q_) values ≤ 38.5 in duplicate were considered positive. Infection rates were calculated using the minimum infection rate (MIR), obtained by dividing the number of positive pools by the total number of mosquitoes analyzed and multiplying the result by 1,000 [36].

### Evaluation of the toxicity of ivermectin before field implementation of the TSB prototype

A laboratory assay was conducted to assess the efficacy of ivermectin dissolved in a sugar solution in inducing adult mosquito mortality after ingestion.

Adult *Ae. aegypti* mosquitoes from the RecL colony of IAM–FIOCRUZ [37], aged 1–3 days, were maintained under controlled conditions (25 ± 1 °C; 50–60% relative humidity; 12:12 h light/dark cycle). Groups of mosquitoes, including 15 males, 15 NBF, and 15 BF, were independently exposed to cotton pads soaked in a 20% sucrose solution containing ivermectin at three concentrations (0.025%, 0.05%, and 0.1%). Negative control groups received a 20% sucrose solution without ivermectin. Each treatment, including the control, was performed in triplicate. Mortality was recorded 12 and 24 h post-exposure. The ivermectin concentration that produced the highest mortality in the shortest time was selected for subsequent field applications.

### Statistical analysis

The following entomological variables were evaluated: (1) the density of *Ae. aegypti* and *Cx. quinquefasciatus* females, and (2) their blood-feeding status (NBF vs. BF). Generalized linear mixed models (GLMMs) with a negative binomial distribution were fitted to assess the effect of the intervention on the number of *Ae. aegypti* females. The first model considered three phases of the intervention (pre-ICP, ICP, and post-ICP). The second model assessed effectiveness across six periods defined by intervention cycles (one pre-intervention period, three intervention periods, and two post-intervention periods). In both models, the pre-intervention data were used as the reference category. Entomological effectiveness was calculated as 1 − IRR, where IRR represents the incidence rate ratio estimated by the negative binomial model. The models provided IRR estimates with their respective 95% confidence intervals, reflecting the proportional reduction in female density compared to the reference period.

For the comparison of qualitative variables related to viral detection (classified as positive or negative), the Chi-square (*χ*^2^) test was employed. Relationships between viral detection positivity, species, and study periods were further explored using multiple correspondence analysis (MCA). All statistical tests were performed with a significance level of *P* < 0.05 in R software version 4.3.1.

## Results

### Efficacy of ivermectin concentrations in inducing adult mosquito mortality

In laboratory assays, all three ivermectin concentrations tested induced high mortality of *Ae. aegypti* adults within 24 h. Among them, the 0.05% and 0.1% concentrations produced the most rapid and consistent lethality, resulting in complete mortality (100%) of males and NBF females and 98% mortality of BF females within 12 h.

To rationalize compound use for field application, the 0.05% concentration was selected, as it produced faster and more uniform mortality than the 0.025% concentration, which caused 36.7% mortality at 12 h and 80% at 24 h among tested adults. This rapid and consistent lethality was considered a key factor for subsequent field deployment.

### Assessment of mosquito populations throughout the study

Baseline sampling during the pre-ICP revealed high levels of *Ae. aegypti* infestation at the HC, with a total of 5802 specimens captured, of which 88% were females, and 65% of them were BF. At the same time the presence of *Cx. quinquefasciatus* was also recorded, with 645 adults captured, showing a similar proportion of females (80%) and BF females (55%). Both species were captured at all 14 stations.

Table 2 shows a consistent and progressive reduction in the adult population of both species following the implementation of the ICP. Adult densities decreased by approximately 70% after 6 months, 90% after 12 months, and more than 99% after 18 months, with a significant impact on *Ae. aegypti* female density compared with the pre-ICP (*P* < 0.001). At the same time, marked reductions in both BF and NBF females was confirmed from 6 months onwards (71%). Although female abundance decreased substantially, the target species remained widely distributed across capture stations up to 12 months after the initiation of integrated control measures. After 18 months of ICP implementation, the spatial distribution of target species declined in nearly half of the capture stations. Five of the 14 capture stations remained positive for mosquitoes, with higher frequencies of *Ae. aegypti* recorded in Reception 4, Hallways, laboratory, Pediatrics, Cardiology and Obstetrics and *Cx. quinquefasciatus* at Receptions 1 and 4 and Obstetrics sector (Table 3).

**Table 2:** Numbers of captured female *Aedes aegypti* and *Culex quinquefasciatus* (including BF individuals) and station positivity for mosquito presence during different periods of the Integrated Control Program (August 2018 to March 2021) at the Hospital das Clínicas, Pernambuco, Brazil

Period	Month/year (time point)	Species	No. of captured females			No. of positive capture stations	Vector control impact
			Total	NBF	BF		
Pre-ICP	Aug 2018	*Ae. aegypti*	5121	1774	3347	14	NA
		*Cx. quinquefasciatus*	516	231	285	14	NA
ICP	Feb 2019 (6 months)	*Ae. aegypti*	1493	623	870	14	71%
		*Cx. quinquefasciatus*	620	420	200	14	0
	Aug 2019 (12 months)	*Ae. aegypti*	534	26	508	14	90%
		*Cx. quinquefasciatus*	250	73	177	14	
	Feb 2020^a^ (18 months)	*Ae. aegypti*	13	6	7	6	> 99%
		*Cx. quinquefasciatus*	62	31	31	10	88%
Post-ICP	Oct 2020^a^ (8 months)	*Ae. aegypti*	41	26	18	11	99%
		*Cx. quinquefasciatus*	105	68	37	14	80%
	Feb 2021^a^ (12 months)	*Ae. aegypti*	52	33	21	13	99%
		*Cx. quinquefasciatus*	81	24	39	13	85%

^a^All captured females were analyzed for NS1 protein by ELISA; NA: Not Analyzed

**Table 3:** Positive capture stations for infected *Aedes aegypti* and *Culex quinquefasciatus* across different locations at the Hospital das Clínicas, Pernambuco, Brazil, during the integrated control program (August 2018 to February 2021)

Period	Month/year	Positive capture station by mosquito species and arbovirus	
		*Ae. aegypti*	*Cx. quinquefasciatus*
Pre-ICP^a^	Aug 2018	Reception 4, hallways, laboratory, small surgeries, triage, pediatrics, cardiology, reception 1, nutrition-deposit; nutrition, obstetrics, surgeries, laundry, morgue	Reception 4, hallways, laboratory, small surgeries, triage, pediatrics, cardiology, reception 1, nutrition-deposit; nutrition, obstetrics, surgeries, laundry, morgue
ICP	Feb 2019	Reception 4^2^, hallways^2^, laboratory^1^, small surgeries^2^, cardiology^1^, reception 1^3^, obstetrics^2^, surgeries^1^, laundry^3^	Reception 4^3^, laboratory^3^, nutrition^1^, reception 1^1^, obstetrics^2^
	Aug 2019	Reception 4^1^, small surgeries^1^, pediatrics^1^, cardiology^1^, reception 1^1^, obstetrics^1^	Reception 4^1^, hallways^1^, laboratory^1^, nutrition^1^, reception 1^1^, obstetrics^1^
	Feb 2020	0	0
Post-ICP^a^	Oct 2020	Reception 4, hallways, laboratory, pediatrics cardiology, laundry, morgue	Nutrition, laboratory, obstetrics
	Feb 2021	Reception 4, hallways, laboratory, pediatrics, cardiology, small surgeries, nutrition, laundry	Cardiology, nutrition,

Superscripts indicate the type of positivity: ^1^NS1 protein only, ^2^NS1 protein and RT-qPCR, ^3^RT-qPCR only

^a^During the pre-ICP and post-ICP, only the NS1 protein was tested

Results obtained and the construction of the GLMMs demonstrate the effectiveness of the ICP in suppressing *Ae. aegypti* females. We observed a marked reduction during ICP, with an IRR ≈ 0.08, corresponding to ~ 92% effectiveness relative to the pre-ICP, followed by a decrease in the post-ICP (IRR ≈ 0.008; effectiveness ~ 99%) (Additional File 2: Table S1 and S2).

During the post-ICP phase, mosquito control actions were interrupted for seven consecutive months (March to September 2020) due to the COVID-19 pandemic. Only the larvicidal treatment of the potential breeding sites in underground areas was restarted from October 2020 to February 2021, to maintain the basic PNCD strategy. Monitoring during this period showed that the numbers of *Ae. aegypti* females remained significantly lower (*P* < 0.001) than those observed in the pre-ICP, with values similar to the quantity observed during the ICP (Additional File 1: Table S1).

The analysis of 3,986 female *Ae. aegypti* and *Cx. quinquefasciatus* collected at the HC across different ICP phases focused on their blood-feeding status and its relationship to gonotrophic condition. During the pre-ICP, 71% of *Ae. aegypti* females were BF. Among these, 16% were gravid and ready for oviposition, indicating more than 3 days since their last blood meal. Longitudinal assessment throughout the ICP showed a continuous decline in the category of females with more than 3 days post-feeding, until this group was no longer detected in October 2019 (ICP). In January 2020, a slight increase in the number of females with more than 3 days since the last blood meal was observed; however, this value later decreased again, and this category was not recorded in the samples from the post-ICP.

For *Cx. quinquefasciatus*, 64% of females were BF during pre-ICP, and 8% had more than 3 days since their last blood meal. Despite the progressive decrease in the total number of females during the ICP and post-ICP, unlike what was observed for *Ae. aegypti*, these categories continued to be detected at percentages similar to those recorded in the pre-ICP. Nevertheless, the marked reduction in adult mosquito density throughout the program suggests a decrease in mosquito–human contact.

### Impact of the ICP on arbovirus detection in female mosquitoes

In the pre-ICP, 50 pools of 10 females were analyzed using only the ELISA assay, as more than 5,000 specimens were collected, and the high sample volume in the entomological bags made it difficult to quickly sort and store the material at −80 °C, leading to viral RNA degradation. NS1-positive females were detected in 12 of the 14 capture stations at this time.

Mosquito samples obtained at the hospital during ICP were analyzed at three time points as follows: 10% of the samples at 6 months, 30% at 12 months, and 100% at 18 months. These different thresholds were established due to the sharp reduction in the number of females captured over the evaluation period. A total of 1,523 mosquitoes were distributed into 254 pools and screened by ELISA, including 137 pools of *Ae. aegypti* and 117 of *Cx. quinquefasciatus*. Among the *Ae. aegypti* pools, 15 tested positive, of which 41% were BF females, while for *Cx. quinquefasciatus*, 16 pools were positive and 81% were BF females (Table 4).

**Table 4:** Number of analyzed female *Aedes aegypti* and *Culex quinquefasciatus* in pools, with corresponding positivity for NS1 and RT-qPCR and minimum infection rate (MIR), during different periods of the integrated control program (August 2018 to February 2021) at the Hospital das Clínicas, Pernambuco, Brazil

Period	Month/year (time point)	Species	Number of analyzed females (positives)		Number of analyzed pools (positives)						MIR	
					Total		NBF		BF			
			NS1	RT-qPCR	NS1	RT-qPCR	NS1	RT-qPCR	NS1	RT-qPCR	NS1	RT-qPCR
Pre-ICP	Aug 2018	*Ae. aegypti*	500 (250)	NA	50 (25)	NA	35 (16)	NA	15 (9)	NA	50	NA
		*Cx. quinquefasciatus*	150 (40)	NA	15 (4)	NA	5 (1)	NA	10 (3)	NA	27	NA
ICP	Feb 2019 (6 months)	*Ae. aegypti*	166 (72)	116 (51)	23 (8)	18 (8)	15 (5)	3 (1)	8 (3)	15 (7)	48	69
		*Cx. quinquefasciatus*	121 (31)	51 (9)	18 (4)	11 (3)	3 (1)	1 (0)	15 (3)	10 (3)	33	59
	Aug 2019 (12 months)	*Ae. aegypti*	159 (37)	109 (6)	24 (7)	19 (1)	8 (5)	16 (1)	16 (2)	3 (0)	44	9
		*Cx. quinquefasciatus*	94 (34)	94 (0)	27 (12)	27 (0)	15 (1)	15 (0)	12 (11)	12 (0)	128	0
	Feb 2020 (18 months)	*Ae. aegypti*	13 (0)	13 (2)	6 (0)	6 (0)	4 (0)	4 (0)	2 (0)	2 (0)	0	0
		*Cx. quinquefasciatus*	62 (0)	62 (0)	15 (0)	15 (0)	8 (0)	8 (0)	7 (0)	7 (0)	0	0
Post-ICP	Oct 2020 (8 months)	*Ae. aegypti*	41 (28)	NA	14 (8)	NA	5 (5)	NA	9 (3)	NA	195	NA
		*Cx. quinquefasciatus*	103 (28)	NA	23 (4)	NA	12 (1)	NA	11 (3)	NA	39	NA
	Feb 2021 (12 months)	*Ae. aegypti*	52 (27)	NA	20 (10)	NA	9 (3)	NA	11 (7)	NA	192	NA
		*Cx. quinquefasciatus*	62 (6)	NA	19 (3)	NA	9 (1)	NA	10 (2)	NA	48	NA

NA: Not Analyzed

Overall, NS1-positive females were detected in nine of the 14 capture stations, indicating widespread local viral circulation. Temporal analysis of NS1 antigen detection between pre-ICP and Aug 2019 (1 year after the ICP implementation) revealed a reduction in the MIR from 50 to 44 and a significant reduction (*χ*^2^ = 4.21, *df* = 1, *P* = 0.04) in pool positivity for *Ae. aegypti*. In contrast, *Cx. quinquefasciatus* showed an increase in MIR. Analyses conducted in February 2020 (ICP) demonstrated that 100% of samples from both species were negative, resulting in MIR = 0.

Regarding RT-qPCR analysis, in total, 167 mosquitoes were collected in February 2019 (ICP), of which 116 were *Ae. aegypti* and 51 *Cx. quinquefasciatus*. From the 29 pools analyzed, 11 pools tested positive exclusively for ZIKV, eight from *Ae. aegypti* and three from *Cx. quinquefasciatus*, the majority represented by BF females (Additional File 1: Table S2). At this same time point, the mean C_q_ value for *Ae. aegypti* was 35.69 (± 1.35), and for *Cx. quinquefasciatus* it was 35.92 (± 0.90), corresponding to MIRs of 69 and 59, respectively. These positive pools were derived from samples collected in reception 4, hallways, small surgeries, and the obstetrics sector.

In August 2019 (12 months after ICP), only one pool composed of two NBF *Ae. aegypti* females, captured in the pediatrics sector, tested positive for DENV, by both ELISA and RT-qPCR. At this time point, the MIR decreased to 9, indicating a low rate of infected females in the hospital environment. No pools tested positive for CHIKV throughout the entire study.

At the final ICP evaluation in February 2020, all tested pools from both species were negative for DENV, ZIKV, and CHIKV, suggesting the mitigation in viral circulation. Over the study period, a progressive decline in the proportion of NBF females was observed, occurring concomitantly with a reduction in infection rates.

Pools positive for ZIKV and DENV by RT-qPCR confirmed viral circulation in the hospital complex at distinct time points. In parallel, NS1 ELISA suggested co-circulation of these arboviruses in at least six capture stations: reception 1 and 4, small surgeries, pediatrics, cardiology, and obstetrics.

Representative samples collected during the post-ICP showed that, although the number of females remained low, MIR values calculated from NS1-positive pools increased (192 and 195) for *Ae. aegypti* compared with pre-ICP and ICP. In contrast, MIR variation for *Cx. quinquefasciatus* was lower, particularly between pre-ICP and post-ICP, and most positive samples from this species were derived from BF females.

**Fig. 3:**
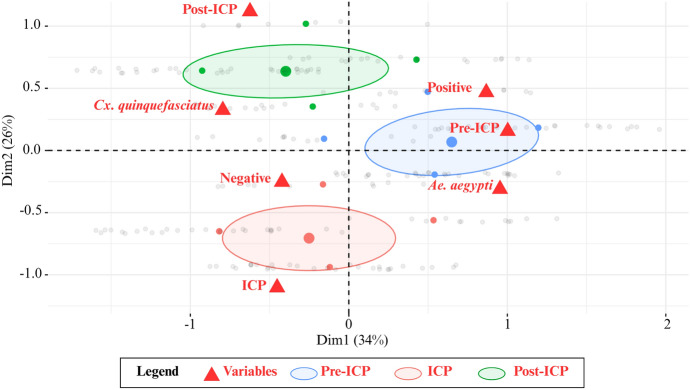
Multiple correspondence analysis of qualitative variables on detection positivity, species, and study periods for *Aedes aegypti* and *Culex quinquefasciatus* captured in the Hospital das Clínicas, Pernambuco, Brazil, during the integrated control program (ICP) from August 2018 to February 2021

Viral detection was also observed in pools composed of only one or two mosquitoes. Using RT-qPCR, ZIKV was detected in one pool of *Cx. quinquefasciatus* (BF) and in two pools of *Ae. aegypti*, one BF and the other NBF. Using NS1 ELISA, we recorded positivity in nine pools of *Ae. aegypti* and the same number of *Cx. quinquefasciatus*, all composed of only one female (BF or NBF). These results became more frequent in samples collected 12 months after the ICP implementation, when the population density of both species had reduced by approximately 90%. However, no overlap was observed between NS1-positive and RT-qPCR-positive samples, except for one of the 24 *Ae. aegypti* samples and one of the 19 *Cx. quinquefasciatus* samples, after strong elimination pressure exerted on the mosquito populations. The confidence ellipse graphic revealed lower overlap as well between the pre-ICP and post-ICP groups, while the ICP group remained more isolated, reflecting distinct patterns across the study phases. Moreover, positive samples were more closely associated with the pre-ICP and post-ICP groups, whereas negative samples were predominantly related to the ICP group (Fig. 3).

## Discussion

The present study demonstrated a scenario of high risk for nosocomial transmission of ZIKV and DENV in a tertiary hospital in Recife and its mitigation as a result of the effectiveness of integrated control actions, which eliminated 99% of adult *Ae. aegypti* mosquitoes in less than 2 years. These findings highlight healthcare facilities as strategic and previously underrecognized hotspots for arbovirus transmission in urban endemic settings and underscore the urgent need to prioritize hospitals in vector surveillance and control policies.

During the pre-ICP, NS1 antigen was detected in approximately 50% of the analyzed pools across nearly all capture stations, indicating widespread circulation of infected females within the hospital environment. These data indicate circulation within both the vector population and the human population occupying the same space. This situation is in line with the historical pattern of increased dengue cases. Official surveillance records indicate that 1,851 arbovirus cases were reported in 2018, 5,164 in 2019, 3,780 in 2020, likely underestimated to disruptions related to COVID-19 and 23,402 in 2021, with dengue accounting for approximately 50–80% of reported cases during this period [38]. Consistently, at the beginning of the study we observed high densities of *Ae. aegypti*, characterizing a highly favorable scenario for arbovirus transmission.

Furthermore, interestingly, the number of *Ae. aegypti* females was approximately 10 times that of *Cx. quinquefasciatus*. This pattern contrasts with findings frequently observed in residential settings of Recife and other municipalities from RMR, where *Cx. quinquefasciatus* consistently predominates over *Ae. aegypti* [21, 39]. This pattern in healthcare facilities may be related to the abundance of suitable temporary breeding sites for *Ae. aegypti* around the HC and the continuous circulation of human hosts in this kind of setting. In contrast, the permanent breeding sites of *Cx. quinquefasciatus* such as canals, domestic sewage systems or street drains [40, 41], and drainage tanks, although present in the hospital surroundings, were not fully covered by the ICP. Over time, the differences in proportion between species were progressively reduced, especially after the implementation of targeted actions to remove or eliminate breeding sites and adult *Ae. aegypti*. Despite peri-hospital conditions that favor continuous reinfestation of the treated area, these actions were sufficient to ensure the continued suppression of *Ae. aegypti* populations, almost eliminating human–vector contact. A comparable focal protection effect was reported by Melo et al. [39], who demonstrated significant reductions in *Ae. aegypti* infestation in highly infested households in Recife after seven months of integrated use of TSB_Ivermec_, adult aspiration, and ovitraps.

We further demonstrated that broader environmental management actions and regular larvicidal treatments of the tanks used for draining water in the HC also contributed to control for both species. This approach demonstrated that it is possible to prevent the presence of all mosquito species in hospital environments, thus mitigating the vulnerability of these places, corroborating the findings of other studies [42, 43].

A detailed analysis of female mosquitoes revealed that the majority of *Ae. aegypti* had successfully taken blood meals and exhibited varying degrees of blood digestion (≤ 3 days and ≥ 3 days) and ovarian development. Throughout the ICP, there was a rapid decline in females with longer post-feeding digestion times (> 3 days), suggesting that control actions preferentially eliminated older, potentially infectious individuals. These findings highlight the importance of incorporating age-grading methods into entomological surveillance to better evaluate the epidemiological impact of vector control strategies in high-risk settings. The physiological age of the captured females shows us that direct age-grading methods must be integrated to evaluate the impact of control measures in similar epidemiological scenarios. Studies confirm that *Ae. aegypti* females with 3 or more days after ingesting an infectious meal with ZIKV, CHIKV, or DENV could release active viral particles in saliva [44–46], therefore beginning to transmit these pathogens. Our findings corroborate those of Traore et al. [47], using the control model *Anopheles gambiae*/TSB, that reported the progressive elimination of older females with up to three oviposition cycles, and at the same time, a reduction in the infection rate of *Plasmodium falciparum* sporozoites in areas of malaria transmission in Mali, Africa. In our study, besides TSB_Ivermec_, the capture of adult mosquitoes by aspiration also enhanced the rapid elimination of females, sometimes before they had a chance to take up a new blood meal.

A study conducted by Melo et al. [39] in Recife, which compared mosquito control in two groups of 20 properties using ovitraps and aspiration with and without the incorporation of TSB_Ivermec_, showed after 1 year a greater and more sustained reduction in *Ae. aegypti* (98%) in properties with TSB_Ivermec_. Together with findings from other studies, these results indicate that integrated control strategies incorporating TSB have strong potential to reduce the risk of arbovirus transmission [39, 48]. Dias et al. [49] and Tenywa et al. [50] also demonstrated TSB as a promising tool for blocking vectorial transmission. According to Foster [51], newly emerged *Ae. aegypti* females are equally likely to choose a sugar meal or a blood meal when both sources are available. This behavior reinforces the strategic value of TSB_Ivermec_ in our study, as it targets both NBF and BF females.

In the pre-ICP, detection of the NS1 antigen in 50% of the analyzed pools and from almost all capture stations revealed the widespread circulation of infected females within the hospital. The presence of these females in healthcare settings reinforces concerns about nosocomial transmission of arboviruses around the world [13, 43, 52, 53], suggesting that these places may act as points for retransmission of DENV and ZIKV. Consistent with these global concerns, our findings showed that after the first 6 months of ICP implementation, females positive to NS1 and RT-qPCR were detected in seven and six of the 14 capture stations, respectively, for *Ae. aegypti*, suggesting a reduction in the risk of vectorial transmission in 50% of the areas treated at the HC. In the 12 months following the ICP, this pattern remained unchanged by the NS1 method, but by RT-qPCR only one pool of NBF females was found positive for DENV in the pediatrics sector.

The C_q_ values obtained in our study (33.82–37.53) were similar to those reported by Krokovsky et al. [13] for both *Ae. aegypti* and *Cx. quinquefasciatus* captured in the HC. This similarity indicated the persistence of ZIKV circulation in these mosquito populations over time and demonstrated the high sensitivity of the molecular methods employed, capable of detecting infection even in mosquitoes with low viral loads.

Nearly 60% of *Ae. aegypti* females that tested positive for arboviruses were classified as NBF. The detection of viral RNA in NBF females raises important questions about possible mechanisms of infection. One hypothesis is that some of those could have completed a gonotrophic cycle prior to capture, having already digested a previous blood meal and initiated oogenesis, which would also explain the absence of visible blood in the abdomen. Another possibility is the vertical transmission, previously documented for several arboviruses including DENV, ZIKV, and CHIKV [54–57]. Similar reasoning could be applied to *Cx. quinquefasciatus.* Further studies combining molecular detection and ovarian dissection analyses are necessary to clarify these routes and assess their epidemiological relevance.

The probability of capturing infected females also decreased, highlighting the importance of sustained entomological interventions to control arbovirus transmission in healthcare settings, considering that the behavior of feeding on blood and sugar occurs continuously throughout the entire life of female mosquitoes [58]. Additionally, the temporal pattern observed in our dataset shows that the reduction in MIR occurred in parallel with an increase in females classified in early blood-meal digestion stages.

In the present study, ZIKV and DENV detection by RT-qPCR were restricted to a limited number of pools and could not be applied to pre-ICP samples due to viral RNA degradation. The difficulty in maintaining sample quality during the pre-ICP period was mainly due to the high number of BF females and the time required for sorting and storage at −80 °C, which likely compromised RNA integrity. These limitations highlight the dependence on rapid processing and cold-chain maintenance as key barriers to the routine use of RT-qPCR in entomological surveillance, as reported in previous studies [26, 59].

The observed differences between the two methodologies in our results highlight the difficulty of working with field-caught mosquitoes. Although RT-qPCR remains the gold standard for virus detection, the NS1 antigen assay demonstrated the potential to serve as a complementary tool for early arboviral surveillance. The integration of systems, such as Flinders Technology Associates (FTA^®^) cards, could enable the detection of arboviruses secreted by mosquito saliva, eliminating the need to deep-refrigerate the samples collected in the field, making it a promising tool for entomo-virological surveillance [60, 61].

The spatial distribution of positive mosquito pools revealed that reception 1 and 4 stations showed higher positivity rates. Although located indoors, these areas remain structurally connected to external environments and are characterized by intense human circulation, which may facilitate mosquito entry and the establishment of focal transmission hotspots. The elevated positivity rates observed in these areas suggest greater entomological pressure from outdoor sources, reinforcing the importance of perimeter-based vector control and physical barriers to prevent mosquito intrusion [62, 63]. Taking this into consideration, physical barriers such as window screens and protective netting are recommended to reduce mosquitoes [64]. In healthcare settings, WHO [65] recommends the use of heating, ventilation, and air conditioning (HVAC) systems to remove contaminated air and support air-handling processes that protect patients and staff from airborne pathogens these systems can also function as a complementary physical barrier to prevent mosquitoes from access hospital environments through doors or windows. When combined with environmental management and routine vector control, these structural measures could improve protection against vector-borne pathogens [64].

Particularly concerning was the detection of ZIKV-positive *Ae. aegypti* and *Cx. quinquefasciatus* females in the obstetrics sector, an area dedicated to high-risk pregnancies and postpartum care. The presence of infected vectors in this location raises important concerns due to the established association between this virus and infection during pregnancy and adverse fetal outcomes, including congenital anomalies [6]. Mapping these hotspots provided an operational framework for prioritizing vector control, focusing on the use of TSB_Ivermec_ and aspiration. From our perspective, this is particularly relevant for hospital sectors that host more vulnerable populations, such as maternal and neonatal care units.

Furthermore, the detection of infected *Cx. quinquefasciatus* females raises additional questions regarding their role as a ZIKV vector as confirmed by some studies [13, 39, 45, 66, 67] and refuted by others [68, 69]. The detection of NS1 antigen in *Cx. quinquefasciatus*, observed here, does not necessarily imply an active infection or true vector competence for DENV, as they are generally unable to sustain DENV replication [70, 71]. In this context, NS1 detection may function as a sentinel marker for the presence of human patients in the viremic period.

Although the observed decline in natural infection rates in *Ae. aegypti* might suggest a mitigation in viral transmission, this decrease is more plausibly attributable to reduced mosquito abundance resulting from intensified integrated control interventions. Beyond mosquitoes, other vector species commonly present in hospital environments, such as flies, also appeared to be affected by the implemented control measures. In our study, an empirical reduction in fly density was observed at the HC following the implementation of ITA_Ivermec_, suggesting that this intervention may exert broader effects beyond its primary target species. This finding corroborates recent evidence reported by [72], which demonstrated the effectiveness of ITA_Ivermec_-based interventions in reducing fly populations using the same ivermectin concentration (0.05%), thereby reinforcing the potential of this approach within integrated vector control strategies in healthcare settings. Reduced vector densities decreased the probability of detecting infected specimens, even when low-level viral circulation persists in the human population. Consequently, the temporal reduction in positive pools should be interpreted with caution, as it likely reflects a combined effect of vector suppression and seasonal variation in arbovirus transmission dynamics.

Finally, our results confirm that both diagnostic approaches were capable of detecting infected pools containing a single female, whether BF or NBF, underscoring their sensitivity and suitability for monitoring temporary or sustained mitigation of the vectorial transmission potential of ZIKV and DENV by *Ae. aegypti* within the HC.

## Conclusions

We provide robust evidence that integrated vector control strategies can effectively suppress *Ae. aegypti* populations and mitigate the risk of nosocomial arbovirus transmission. Combined strategies including TSB_Ivermec_ and the mechanical elimination of adults and eggs provided efficient and sustainable control, offering an alternative to the application of conventional chemical insecticides in healthcare settings. At the same time, the integration of entomo-virological surveillance proved to be a powerful strategy for detecting the circulation of arboviruses within the hospital. In this context, NS1 ELISA could serve as a rapid cost-effective screening tool, complementing RT-qPCR assays. The findings of our study establish an integrated framework for mitigating arbovirus transmission in hospital environments and support its adoption as a strategic model for protecting patients and healthcare employees in HC and other SUS healthcare units.

## Supplementary Information

Additional file 1.

Additional file 2.

Additional file 3.

## Data Availability

Raw data from all assays are available in the supplementary tables.

## Abbreviations

BF: Blood-fed
Bti: *Bacillus thuringiensis israelensis*
CAL: Calibrator
CHIKV: Chikungunya virus
CZS: Congenital Zika syndrome
C_q_: Quantification cycle
DENV: Dengue virus
ELISA: Enzyme-linked immunosorbent assay
FBS: Fetal bovine serum
GLMMs: Generalized Linear Mixed Models
HC: Hospital das Clínicas
ICP: Integrated control program
IRR: Incidence Rate Ratio
MIR: Minimum infection rate
NBF: Non-blood-fed
NS1: Nonstructural protein 1
OROV: Oropouche virus
PNCD: Programa Nacional de Controle da Dengue
PVC: Polyvinyl chloride
RMR: Recife Metropolitan Region
RT-qPCR: Reverse transcription quantitative polymerase chain reaction
SUS: Sistema Único de Saúde
TSB: Toxic sugar bait
TSB_Ivermec_: Toxic sugar baits treated with ivermectin
UBS: Unidade Básica de Saúde
UPA: Unidade de Pronto Atendimento
UFPE: Universidade Federal de Pernambuco
