# Supplementary material for: Control and mitigation of dengue and Zika virus transmission in a hospital in Recife, Brazil: a successful experience with an integrated control program against Aedes aegypti
Source: Parasit Vectors. 2026 Mar 13;19:174. doi: 10.1186/s13071-025-07241-9 (PMC13097936; doi:10.1186/s13071-025-07241-9)
Supplement: Supplementary file 1 — Additional file 1. [file 13071_2025_7241_MOESM1_ESM.docx]

Additional File 1: Table S1. Characteristics of positive mosquito pools tested by ELISA NS1 DENV antigen.

| Month of Collection | Sample Name | Species | Number of females | Physiological Status | Capture Station |
| --- | --- | --- | --- | --- | --- |
| Aug/18 | Sample 1 | *A. aegypti* | 10 | BF | General |
| Aug/18 | Sample 2 | *A. aegypti* | 10 | BF | General |
| Aug/18 | Sample 4 | *A. aegypti* | 10 | BF | General |
| Aug/18 | Sample 6 | *A. aegypti* | 10 | BF | General |
| Aug/18 | Sample 7 | *A. aegypti* | 10 | BF | General |
| Aug/18 | Sample 10 | *A. aegypti* | 10 | BF | General |
| Aug/18 | Sample 12 | *C. quinquefasciatus* | 10 | BF | General |
| Aug/18 | Sample 15 | *C. quinquefasciatus* | 10 | BF | General |
| Aug/18 | Sample 16 | *C. quinquefasciatus* | 10 | BF | General |
| Aug/18 | Sample 377 | *A. aegypti* | 10 | BF | General |
| Aug/18 | Sample 379 | *A. aegypti* | 10 | BF | General |
| Aug/18 | Sample 381 | *A. aegypti* | 10 | BF | General |
| Aug/18 | Sample 382 | *A. aegypti* | 10 | UF | General |
| Aug/18 | Sample 387 | *A. aegypti* | 10 | UF | General |
| Aug/18 | Sample 388 | *A. aegypti* | 10 | UF | General |
| Aug/18 | Sample 389 | *A. aegypti* | 10 | UF | General |
| Aug/18 | Sample 391 | *A. aegypti* | 10 | UF | General |
| Aug/18 | Sample 393 | *A. aegypti* | 10 | UF | General |
| Aug/18 | Sample 395 | *A. aegypti* | 10 | UF | General |
| Aug/18 | Sample 396 | *A. aegypti* | 10 | UF | General |
| Aug/18 | Sample 398 | *A. aegypti* | 10 | UF | General |
| Aug/18 | Sample 399 | *A. aegypti* | 10 | UF | General |
| Aug/18 | Sample 400 | *A. aegypti* | 10 | UF | General |
| Aug/18 | Sample 401 | *A. aegypti* | 10 | UF | General |
| Aug/18 | Sample 403 | *A. aegypti* | 10 | UF | General |
| Aug/18 | Sample 404 | *A. aegypti* | 10 | UF | General |
| Aug/18 | Sample 414 | *A. aegypti* | 10 | UF | General |
| Aug/18 | Sample 416 | *A. aegypti* | 10 | UF | General |
| Aug/18 | Sample 423 | *C. quinquefasciatus* | 10 | UF | General |
| Feb/19 | 13/19-02 | *C. quinquefasciatus* | 1 | BF | Obstetrics |
| Feb/19 | 18/19-01 | *A. aegypti* | 10 | BF | Central corridors |
| Feb/19 | 19/19-01 | *A. aegypti* | 10 | BF | Laboratory/Triage |
| Feb/19 | 20/19-01 | *C. quinquefasciatus* | 10 | BF | Nutrition |
| Feb/19 | 22/19-01 | *A. aegypti* | 2 | UF | Small surgeries |
| Feb/19 | 24/19-01 | *A. aegypti* | 10 | BF | Pediatrics |
| Feb/19 | Sample 365 | *A. aegypti* | 10 | UF | Reception 4 |
| Feb/19 | Sample 367 | *A. aegypti* | 10 | UF | Laboratory/Triage |
| Feb/19 | Sample 368 | *A. aegypti* | 10 | UF | Surgeries |
| Feb/19 | Sample 369 | *A. aegypti* | 10 | UF | Cardiology |
| Feb/19 | Sample 370 | *C. quinquefasciatus* | 10 | UF | Reception 1 |
| Feb/19 | Sample 372 | *C. quinquefasciatus* | 10 | BF | Nutrition |
| Aug/19 | 60/19-01 | *A. aegypti* | 2 | UF | Pediatrics |
| Aug/19 | 62/19-01 | *C. quinquefasciatus* | 2 | BF | Nutrition |
| Aug/19 | 64/19-03 | *C. quinquefasciatus* | 2 | BF | Central corridors |
| Aug/19 | 65/19-02 | *C. quinquefasciatus* | 2 | BF | Reception 4 |
| Aug/19 | 66/19-01 | *C. quinquefasciatus* | 4 | BF | Obstetrics |
| Aug/19 | 68/19-01 | *A. aegypti* | 3 | UF | Reception 1 |
| Aug/19 | 69/19-01 | *A. aegypti* | 6 | UF | Pediatrics |
| Aug/19 | 70/19-01 | *C. quinquefasciatus* | 3 | BF | Nutrition |
| Aug/19 | 72/19-01 | *C. quinquefasciatus* | 1 | BF | Laboratory/Triage |
| Aug/19 | 73/19-04 | *C. quinquefasciatus* | 1 | BF | Central corridors |
| Aug/19 | 74/19-03 | *C. quinquefasciatus* | 1 | BF | Reception 4 |
| Aug/19 | 75/19-01 | *A. aegypti* | 3 | UF | Obstetrics |
| Aug/19 | 77/19-01 | *C. quinquefasciatus* | 1 | BF | Reception 1 |
| Aug/19 | 79/19-01 | *C. quinquefasciatus* | 4 | BF | Nutrition |
| Aug/19 | 80/19-01 | *A. aegypti* | 3 | UF | Small surgeries |
| Aug/19 | 82/19-02 | *C. quinquefasciatus* | 3 | BF | Laundry |
| Aug/19 | Sample 357 | *A. aegypti* | 10 | BF | Reception 4 |
| Aug/19 | Sample 361 | *A. aegypti* | 10 | BF | Cardiology |
| Aug/19 | Sample 362 | *C. quinquefasciatus* | 10 | UF | Obstetrics |
| Oct/20 | Sample 225 | *A. aegypti* | 8 | BF | Reception 4 |
| Oct/20 | Sample 227 | *A. aegypti* | 5 | BF | Central corridors |
| Oct/20 | Sample 228 | *A. aegypti* | 5 | UF | Central corridors |
| Oct/20 | Sample 231 | *C. quinquefasciatus* | 6 | UF | Obstetrics |
| Oct/20 | Sample 232 | *C. quinquefasciatus* | 8 | BF | Nutrition |
| Oct/20 | Sample 316 | *A. aegypti* | 1 | BF | Cardiology |
| Oct/20 | Sample 319 | *A. aegypti* | 5 | UF | Laboratory/Triage |
| Oct/20 | Sample 320 | *A. aegypti* | 1 | UF | Laundry |
| Oct/20 | Sample 321 | *A. aegypti* | 1 | UF | Morgue |
| Oct/20 | Sample 322 | *A. aegypti* | 2 | UF | Pediatrics |
| Oct/20 | Sample 325 | *C. quinquefasciatus* | 10 | BF | Nutrition |
| Oct/20 | Sample 337 | *C. quinquefasciatus* | 4 | BF | Laboratory/Triage |
| Feb/21 | Sample 269 | *A. aegypti* | 1 | UF | Reception 4 |
| Feb/21 | Sample 273 | *A. aegypti* | 6 | UF | Laboratory/Triage |
| Feb/21 | Sample 287 | *A. aegypti* | 2 | BF | Reception 4 |
| Feb/21 | Sample 289 | *A. aegypti* | 2 | BF | Central corridors |
| Feb/21 | Sample 290 | *A. aegypti* | 2 | BF | Pediatrics |
| Feb/21 | Sample 292 | *A. aegypti* | 8 | BF | Laboratory/Triage |
| Feb/21 | Sample 293 | *A. aegypti* | 2 | BF | Cardiology |
| Feb/21 | Sample 295 | *A. aegypti* | 2 | UF | Small Surgeries |
| Feb/21 | Sample 299 | *A. aegypti* | 1 | BF | Laundry |
| Feb/21 | Sample 301 | *A. aegypti* | 1 | BF | Nutrition |
| Feb/21 | Sample 303 | *C. quinquefasciatus* | 3 | BF | Nutrition |
| Feb/21 | Sample 310 | *C. quinquefasciatus* | 1 | BF | Cardiology |
| Feb/21 | Sample 311 | *C. quinquefasciatus* | 2 | UF | Cardiology |

Additional File 1: Table S2. Characteristics of positive mosquito pools tested by triplex RT-qPCR for DENV, ZIKV, and CHIKV, including detected virus per sample, mean Cq values, and mean quantity of RNA copies/mL.

| Month of Collection | Sample Name | Species | Number of females/pool | Physiological Status | Capture Station | Detected Virus | Cq mean | Quantity Mean of RNA copies/ mL |
| --- | --- | --- | --- | --- | --- | --- | --- | --- |
| feb/19 | 11/19-01 | *A. aegypti* | 4 | BF | Reception 1 | ZIKV | 37.3 | 1.03E+07 |
| feb/19 | 13/19-01 | *A. aegypti* | 2 | BF | Obstetrics | ZIKV | 34.2 | 5.78E+07 |
| feb/19 | 13/19-02 | *C. quinquefasciatus* | 1 | BF | Obstetrics | ZIKV | 36.3 | 1.49E+07 |
| feb/19 | 17/19-04 | *A. aegypti* | 10 | BF | Reception 4 | ZIKV | 35.1 | 4.91E+07 |
| feb/19 | 19/19-03 | *C. quinquefasciatus* | 5 | BF | Laboratory/Triage | ZIKV | 35.2 | 5.88E+07 |
| feb/19 | 22/19-01 | *A. aegypti* | 2 | UF | Small Surgeries | ZIKV | 35.2 | 5.42E+07 |
| feb/19 | 23/19-01 | *A. aegypti* | 5 | BF | Obstetrics | ZIKV | 34.2 | 1.50E+08 |
| feb/19 | 27/19-01 | *A. aegypti* | 10 | BF | Reception 4 | ZIKV | 35.6 | 6.04E+07 |
| feb/19 | 27/19-07 | *C. quinquefasciatus* | 3 | BF | Reception 4 | ZIKV | 34.2 | 1.05E+08 |
| feb/19 | 28/19-01 | *A. aegypti* | 10 | BF | Central Corridors | ZIKV | 37.3 | 1.31E+07 |
| feb/19 | 29/19-01 | *A. aegypti* | 8 | BF | Laboratory/Triage | ZIKV | 33.4 | 2.63E+08 |
| aug/19 | 69/19-01 | *A. aegypti* | 6 | UF | Pediatrics | DENV | 28.4 | 8.34E+08 |
