# Supplementary material for: Control and mitigation of dengue and Zika virus transmission in a hospital in Recife, Brazil: a successful experience with an integrated control program against Aedes aegypti
Source: Parasit Vectors. 2026 Mar 13;19:174. doi: 10.1186/s13071-025-07241-9 (PMC13097936; doi:10.1186/s13071-025-07241-9)
Supplement: Supplementary file 2 — Additional file 2. [file 13071_2025_7241_MOESM2_ESM.docx]

Additional File 2: Table S1. GLMM analysis considering the abundance of *Aedes aegypti* during three different periods of the Integrated Control Program (August 2018 to February 2021) at the Hospital das Clínicas, Pernambuco, Brazil.

| Variable | Estimate (log) | IRR | IC95% (IRR) | p-value | Efectivity (1-IRR) |
| --- | --- | --- | --- | --- | --- |
| Intercept (Pre-ICP) | 5.68 | 292.9 | 94.9 – 904.2 | <0.001 | — (reference) |
| ICP vs Pre-ICP | -2.53 | 0.079 | 0.027 – 0.231 | <0.001 | 92.1% |
| Post-ICP vs Pre-ICP | -4.78 | 0.008 | 0.003 – 0.027 | <0.001 | 99.2% |

Additional File 2: Table S2. GLMM analysis considering the abundance of *Aedes aegypti* at different timepoints within the periods of the Integrated Control Program (August 2018 to February 2021) at the Hospital das Clínicas, Pernambuco, Brazil.

| Variable (Month vs Pre-ICP) | Estimate (log) | IRR | IC95% (IRR) | p-value | Efectivity (1-IRR) |
| --- | --- | --- | --- | --- | --- |
| Intercept (Pre-ICP) | 5.51 | 246.9 | 111.5 – 546.9 | <0.001 | — (reference) |
| Feb/2019 vs Pre-ICP | -1.66 | 0.189 | 0.092 – 0.391 | <0.001 | 81.1% |
| Aug/2019 vs Pre-ICP | -2.61 | 0.074 | 0.036 – 0.154 | <0.001 | 92.6% |
| Feb/2020 vs Pre-ICP | -6.04 | 0.002 | 0.001 – 0.006 | <0.001 | 99.8% |
| Oct/2020 vs Pre-ICP | -5.03 | 0.006 | 0.003 – 0.015 | <0.001 | 99.3% |
| Feb/2021 vs Pre-ICP | -4.45 | 0.012 | 0.005 – 0.026 | <0.001 | 98.8% |
