# Supplementary material for: Control and mitigation of dengue and Zika virus transmission in a hospital in Recife, Brazil: a successful experience with an integrated control program against Aedes aegypti
Source: Parasit Vectors. 2026 Mar 13;19:174. doi: 10.1186/s13071-025-07241-9 (PMC13097936; doi:10.1186/s13071-025-07241-9)
Supplement: Supplementary file 3 — Additional file 3. [file 13071_2025_7241_MOESM3_ESM.pdf]

SPRINGER NATURE

Change of authorship request form - Journals (pre-acceptance)

Important information. Please read.

- This form should be used by authors to request any change in authorship (adding/deleting authors) including changes in corresponding authors. This form should not be used for name changes. Please fully complete all sections. Use black ink and block capitals and provide each author's full name with the given name first followed by the family name.

- By signing this declaration, all authors guarantee that the order of the authors are in accordance with their scientific contribution, if applicable as different conventions apply per discipline, and that only authors have been added who made a meaningful contribution to the work.

- Please note, in author collaborations where there is formal agreement for representing the collaboration, it is sufficient for the representative or legal guarantor (usually the corresponding author) to complete and sign the Authorship Change Form on behalf of all authors, **next to the added/removed author(s)**. **(Complete Section 3, followed by Section 6.)**
  In author collaborations where there is no formal agreement for representing the collaboration and **there are more than 10 authors**, one may sign for all, provided the signer appends correspondence that attests that each of the authors have agreed to the change **and the added/removed authors sign the form**. **(Complete Section 3, followed by Section 6.)**

- Please note, we cannot investigate or mediate any authorship disputes. If you are unable to obtain agreement from all authors (including those who you wish to be removed) you must refer the matter to your institution(s) for investigation. Please inform us if you need to do this.

- If you are not able to return a fully completed form within **30 days** of the date that it was sent to the author requesting the change, we may have to withdraw your manuscript. We cannot publish manuscripts where authorship has not been agreed by all authors (including those who have been removed).

- Incomplete forms will be rejected.

- Please return/upload this form, fully completed, to the Journals Editorial Office. The Journal and/or Publisher will consider the information you have provided to decide whether to approve the proposed change in authorship. We may decide to contact your institution for more information or undertake a further investigation, if appropriate, before making a final decision.

SPRINGER NATURE Springer Nature is one of the world's leading global research, educational and professional publishers, created in May 2015 through the combination of Nature Publishing Group, Palgrave Macmillan, Macmillan Education and Springer Science+Business Media.

SPRINGER NATURE

Change of authorship request form - Journals (pre-acceptance)

Section 1: Please provide the current title of manuscript

Manuscript ID no.: 6b95005e-e014-4eba-8d6d-b0598b6be553

Title: Interruption of vector-born circulation of Dengue and Zika Viruses in a hospital from Recife-PE, Brazil: a success experience of the Integrated Control Program against *Aedes aegypti*

Section 2: Please provide the previous authorship, in the order shown on the manuscript before the changes were introduced. Please indicate the corresponding author by adding (CA) behind the name.

	First name(s)	Family name	ORCID or SCOPUS id, if available
1st author	Helena Emanuela	Candida-Silva (CA)	
2nd author	Jaziela de Arruda	Mendonça	
3rd author	Rafael Alves da	Silva	
4th author	Larissa	Krokovsky	
5th author	Henrique Rafael Pontes	Ferreira	
6th author	Kathyanne Ellen da Silva	Barbosa	
7th author	Frederico Jorge	Ribeiro	
8th author	Cláudia Maria Fontes de	Oliveira	
9th author	Constância Flávia Junqueira	Ayres	
10th author	Marcelo Henrique Santos	Paiva	
11th author	Maria Alice Varjal de	Melo-Santos	

Please use an additional sheet if there are more than 10 authors.

SPRINGER NATURE Springer Nature is one of the world's leading global research, educational and professional publishers, created in May 2015 through the combination of Nature Publishing Group, Palgrave Macmillan, Macmillan Education and Springer Science+Business Media.

SPRINGER NATURE

Change of authorship request form - Journals (pre-acceptance)

Section 3: Please provide a justification for change. Please use this section to explain your reasons for changing the authorship of your manuscript, e.g. what necessitated the change in authorship? Please refer to the (journal) policy pages for more information about authorship. Please explain why omitted authors were not originally included and/or why authors were removed on the submitted manuscript.

The graduate program to which the author HECS is affiliated requires that the PI (MAVMS) be listed as the corresponding author; therefore, this change is necessary to comply with institutional regulations. The author LOM has been added because she performed the proof-of-concept experiments and made a meaningful contribution to the work. Additionally, the author HRPF has been reassigned to the position of second author in recognition of his recent contributions to the manuscript development and statistical analyses.

Section 4: Proposed new authorship. Please provide your new authorship list in the order you would like it to appear on the manuscript. Please indicate the corresponding author by adding (CA) behind the name. <u>If the Corresponding Author has changed, please indicate the reason under section 3</u>.

	First name(s)	Family name (this name will appear in full on the final publication and will be searchable in various abstract and indexing databases)	Affiliated institute	E-mail address
1st author	Helena Emanuela	Candida-Silva	Instituto Aggeu Magalhães, Fundação Oswaldo Cruz (FIOCRUZ), Departamento de Entomologia, Recife, Pernambuco, Brazil	manucandido.s@gmail.com
2nd author	Henrique Rafael Pontes	Ferreira	Instituto Aggeu Magalhães, Fundação Oswaldo Cruz (FIOCRUZ), Departamento de Entomologia, Recife, Pernambuco, Brazil	henrique.pontes@ufpe.br
3rd author	Jaziela de Arruda	Mendonça	Instituto Aggeu Magalhães, Fundação Oswaldo Cruz (FIOCRUZ), Departamento de Entomologia, Recife, Pernambuco, Brazil	mjaziela@gmail.com
4th author	Rafael Alves da	Silva	Instituto Aggeu Magalhães, Fundação Oswaldo Cruz (FIOCRUZ), Departamento de Entomologia, Recife, Pernambuco, Brazil	rafaelalvesmicrobiologia2016@gmail.com
5th author	Larissa	Krokovsky	Instituto Aggeu Magalhães, Fundação Oswaldo Cruz (FIOCRUZ), Departamento de Entomologia, Recife, Pernambuco, Brazil	lkrokovsky@gmail.com
6th author	Letícia de Oliveira	Martins	Instituto Aggeu Magalhães, Fundação Oswaldo Cruz (FIOCRUZ), Departamento de Entomologia,	leticiamartins0416@gmail.com

---
SPRINGER NATURE Springer Nature is one of the world's leading global research, educational and professional publishers, created in May 2015 through the combination of Nature Publishing Group, Palgrave Macmillan, Macmillan Education and Springer Science+Business Media.

			Recife, Pernambuco, Brazil	
7th author	Kathyanne Ellen da Silva	Barbosa	Instituto Aggeu Magalhães, Fundação Oswaldo Cruz (FIOCRUZ), Departamento de Entomologia, Recife, Pernambuco, Brazil	kathyannellen@gmail.com
8th author	Frederico Jorge	Ribeiro	Hospital das Clínicas, UFPE/EBSERH, Recife, Pernambuco, Brazil	fredericojor@gmail.com
9th author	Cláudia Maria Fontes de	Oliveira	Instituto Aggeu Magalhães, Fundação Oswaldo Cruz (FIOCRUZ), Departamento de Entomologia, Recife, Pernambuco, Brazil	claudia.fontes@fiocruz.br
10th author	Constância Flávia Junqueira	Ayres	Instituto Aggeu Magalhães, Fundação Oswaldo Cruz (FIOCRUZ), Departamento de Entomologia, Recife, Pernambuco, Brazil	constancia.ayres@fiocruz.br
11th author	Marcelo Henrique Santos	Paiva	Instituto Aggeu Magalhães, Fundação Oswaldo Cruz (FIOCRUZ), Departamento de Entomologia, Recife, Pernambuco, Brazil and Núcleo de Ciências da Vida, Universidade Federal de Pernambuco, Caruaru, Pernambuco, Brazil.	marcelo.paiva@fiocruz.br
12th author	Maria Alice Varjal de	Melo-Santos (CA)	Instituto Aggeu Magalhães, Fundação Oswaldo Cruz (FIOCRUZ), Departamento de Entomologia, Recife, Pernambuco, Brazil	maria.varjal@fiocruz.br

Please use an additional sheet if there are more than 10 authors.

SPRINGER NATURE Springer Nature is one of the world’s leading global research, educational and professional publishers, created in May 2015 through the combination of Nature Publishing Group, Palgrave Macmillan, Macmillan Education and Springer Science+Business Media.

SPRINGER NATURE

Change of authorship request form - Journals (pre-acceptance)

Section 5: Author contribution, Acknowledgement and Disclosures. Please use this section to provide a new disclosure statement and, if appropriate, acknowledge any contributors who have been removed as authors and ensure you state what contribution any new authors made (if applicable per the journal or book (series) policy). Please ensure these are updated in your manuscript - after approval of the change(s) - as our production department will not transfer the information in this form to your manuscript.

New acknowledgements:

We would like to thank the insectary team and the Serviço de Referência em Controle de Culicídeos Vetores (SRCCV) at Fiocruz-PE; José Ribeiro de Lima for support with field collections; Izolda Moura, Danielle Santos and Marileide Brito for assistance with fieldwork at the HC; Verônica Gomes da Silva, Dra. Marly Tenório Cordeiro and Dra. Clarice Neuenschwander Lins de Morais from Serviço de Referência em Arbovírus (SRA) at Virology Department (Fiocruz-PE) for providing the ELISA kits and for training in the technique and Luisa Inácio for help with molecular analyses.

New Disclosures (financial and non-financial interests, funding):

New Author Contributions statement (if applicable per the journal policy):

LOM conducted the experimental assays

State 'Not applicable' if there are no new authors.

SPRINGER NATURE Springer Nature is one of the world's leading global research, educational and professional publishers, created in May 2015 through the combination of Nature Publishing Group, Palgrave Macmillan, Macmillan Education and Springer Science+Business Media.

SPRINGER NATURE

Change of authorship request form - Journals (pre-acceptance)

Section 6: Declaration of agreement. *All* authors, unchanged, new and removed *must* sign this declaration.

(NB: Please print the form, (docu)-sign and return/upload a scanned copy. Please note that signatures that have been inserted as an image file are acceptable as long as it is handwritten. Typed names in the signature box are unacceptable.) *** Please delete as appropriate. Delete all of the bold if you were on the original authorship list and are remaining as an author.**

	First name	Family name		Signature	Date
1st author	Helena Emanuela	Candida-Silva	I agree to the proposed new authorship shown in section 4 and the proposed change in corresponding author	Helena Emanuela Candida-Silva	12/02/2025
2nd author	Henrique Rafael Pontes	Ferreira	I agree to the proposed new authorship shown in section 4 and the proposed change in corresponding author	[illegible]	12/02/2025
3rd author	Jaziela de Arruda	Mendonça	I agree to the proposed new authorship shown in section 4 and the proposed change in corresponding author	Jaziela de Arruda Mendonça	12/11/2025
4th authors	Rafael Alves da	Silva	I agree to the proposed new authorship shown in section 4 and the proposed change in corresponding author	Rafael Alves da Silva	12/02/2025
5th author	Larissa	Krokovsky	I agree to the proposed new authorship shown in section 4 and the proposed change in corresponding author	Larissa Krokovsky	12/11/2025
6th author	Letícia de Oliveira	Martins	I agree to the proposed new authorship shown in section 4 and the addition of my name to the authorship list and the proposed change in corresponding author	Letícia de Oliveira Martins	12/09/2025
7th author	Kathyanne Ellen da Silva	Barbosa	I agree to the proposed new authorship shown in section 4 and the proposed change in corresponding author	Kathyanne Ellen da S. Barbosa	12/03/2025

8th author	Frederico Jorge	Ribeiro	I agree to the proposed new authorship shown in section 4 and the proposed change in corresponding author	Frederico Jorge Ribeiro	12/02/2025
9th author	Cláudia Maria Fontes de	Oliveira	I agree to the proposed new authorship shown in section 4 and the proposed change in corresponding author	CMFOliveira	12/03/2025
10th author	Constância Flávia Junqueira	Ayres	I agree to the proposed new authorship shown in section 4 and the proposed change in corresponding author	Constância Ayres	12/04/2025
11th author	Marcelo Henrique Santos	Paiva	I agree to the proposed new authorship shown in section 4 and the proposed change in corresponding author	Marcelo H. S. Paiva	12/02/2025
12th author	Maria Alice Varjal de	Melo-Santos	I agree to the proposed new authorship shown in section 4 and the proposed change in corresponding author	maria Alice varjal de melo santos	12/02/2025

SPRINGER NATURE Springer Nature is one of the world's leading global research, educational and professional publishers, created in May 2015 through the combination of Nature Publishing Group, Palgrave Macmillan, Macmillan Education and Springer Science+Business Media.

Change of authorship request form - Journals (pre-acceptance)

In case of author collaborations with formal agreement:

	Name of consortium/consortia	First name	Family name		Signature	Date
Representative/legal guarantor				I agree to the proposed new authorship shown in section 4 /**and the addition/removal*of my name to the authorship list** /and the proposed change in corresponding author		

Both added/removed authors should complete the information in the first table under Section 6.

---- End of form ----

SPRINGER NATURE Springer Nature is one of the world's leading global research, educational and professional publishers, created in May 2015 through the combination of Nature Publishing Group, Palgrave Macmillan, Macmillan Education and Springer Science+Business Media.
